# Intracranial dissemination in a primary small cell carcinoma of the brain: a case report and literature review

**DOI:** 10.3389/fonc.2023.1222961

**Published:** 2023-09-13

**Authors:** Yesheng Sun, Ying Zhang, Ruichun Li, Dongpeng Cai, Wei Zhang, Zhiqian Yang

**Affiliations:** ^1^ Department of Neurosurgery, First Affiliated Hospital of Guangdong Pharmaceutical University, Guangzhou, China; ^2^ Guangdong Provincial Engineering and Technology Research Center of Stem Cell Therapy for Pituitary Disease, Guangzhou, China

**Keywords:** primary intracranial small cell carcinoma, metastasis, radiotherapy, chemotherapy, immunohistochemistry

## Abstract

Primary intracranial small cell carcinoma (SCC) is extremely rare with only 8 previously reported cases. We describe a case of primary intracranial SCC with intracranial metastasis. A 46-year-old man presented with decreased vision and a red and swollen left eye. Brain magnetic resonance imaging (MRI) revealed a heterogeneously enhanced tumor on the left frontal lobe. Preoperative systemic computed tomography (CT), MRI, and positron emission tomography (PET)-CT revealed no extracranial tumors. The tumor on the left frontal lobe was excised. Immunohistochemical staining on the excision showed positivity for CD56, synaptophysin (Syn), cytokeratin (CK), and Ki-67 (30%), and negativity for thyroid transcriptional factor-1 (TTF-1), glial fibrillary acidic protein (GFAP), B-cell lymphoma 6 (Bcl-6), multiple myeloma oncogene 1 (MUM-1), C-Myc, Vimentin, P40, P53, CK7, CD3, CD5, CD20, CD79a, CD10, and CD23. The pathological examination strongly suggested that the tumor was a primary intracranial SCC. One year after the surgery, the patient was readmitted with slurred speech and slow movements. Three well-defined tumors were found in the left upper frontal lobe by brain MRI. Tumor resection was then performed. Further immunohistochemical examination of the excised tissue displayed the same pattern as previously, indicating the recurrence of intracranial SCC in the left frontal lobe. The patient received adjuvant chemotherapy and radiotherapy after the tumor resection. At the 2-year follow-up, he remained asymptomatic.

## Introduction

Small cell carcinoma (SCC) is highly aggressive and most commonly arises in the lung. However, extrapulmonary small cell carcinoma (EPSCC) accounts for 2.5% to 4% of all SCC, and can originate from virtually any organ in either pure or mixed form (1). More than 70% of patients with EPSCC are older than 50 years, and the most common primary sites are the gastrointestinal tract and genitourinary system. The histologic criteria for a diagnosis of EPSCC are similar to that of pulmonary neoplasms: round- to spindle-shaped small cells with dense nuclei; sparse cytoplasm; and inconspicuous nucleoli. Necrosis and high mitotic rate are typical (2). Due to the histopathological similarity, it is essential to differentiate newly diagnosed primary EPSCC from metastasized pulmonary tumors.

Brain metastasis is mostly found in small-cell lung cancer (SCLC), accounting for 11% of all SCLC cases. Metastatic brain tumors with the absence of any detectable primary sites are very rare, and primary intracranial SCC is even less so—only 8 cases have been documented in the literature (3–9) (**Table 1**). Here, we describe a case of primary intracranial SCC with intracranial metastasis.

**Table 1:** Review of literature describing primary intracranial SCC.

	Case #	Age, y	Sex	Biomarkers, +	Treatment	Follow-up
Galanis (3)	8	ND	M	ND	Surgery + RT + CTX	ND
Hueser (4)	4	59	F	Syn, TTF-1	Surgery + CTX	31 mo, died
Noonan (5)	5	43	F	Pancytokeratin, EMA, CK7, TTF-1, villin, CEA, CA125	Surgery + RT + CTX	5 y, free
Yang (6)	2	68	M	Syn, TTF-1, CD56, Ki-67,		ND, died
Terada (7)	3	75	M	CK 7, CK 18, CD56, TTF-1, KIT, PDGFRA, NSE, Ki-67, Syn, p53, chromogranin	Surgery + RT + CTX	ND
Zhang (8)	1	69	F	Syn, TTF-1, CgA NSE, CD56, pCK	Surgery + RT	12 mo, free
Chionh (9)	6	56	M	ND	Surgery + RT + CTX	31 mo, free
Chionh (9)	7	71	F	ND	Surgery + RT + CTX	24 mo, died

CgA, chromogranin A; CK, cytokeratin; CTX, chemotherapy; F, female; M, male; ND, not described; NSE, neuron-specific enolase; PDGFRA, platelet-derived growth factor receptor-α; RT, radiotherapy; Syn, synaptophysin; TTF-1, thyroid transcription factor-1.

## Case presentation

A 46-year-old man was referred to our department complaining of a red and swollen left eye, decreased vision, increased tears, and sensitivity to the wind for 2 weeks, accompanied by occasional headaches and mild dizziness. He denied recent eye trauma. The past medical history was not relevant. Ophthalmic examination revealed a swollen, edematous left eyelid with conjunctival congestion.

**Figure 1:**
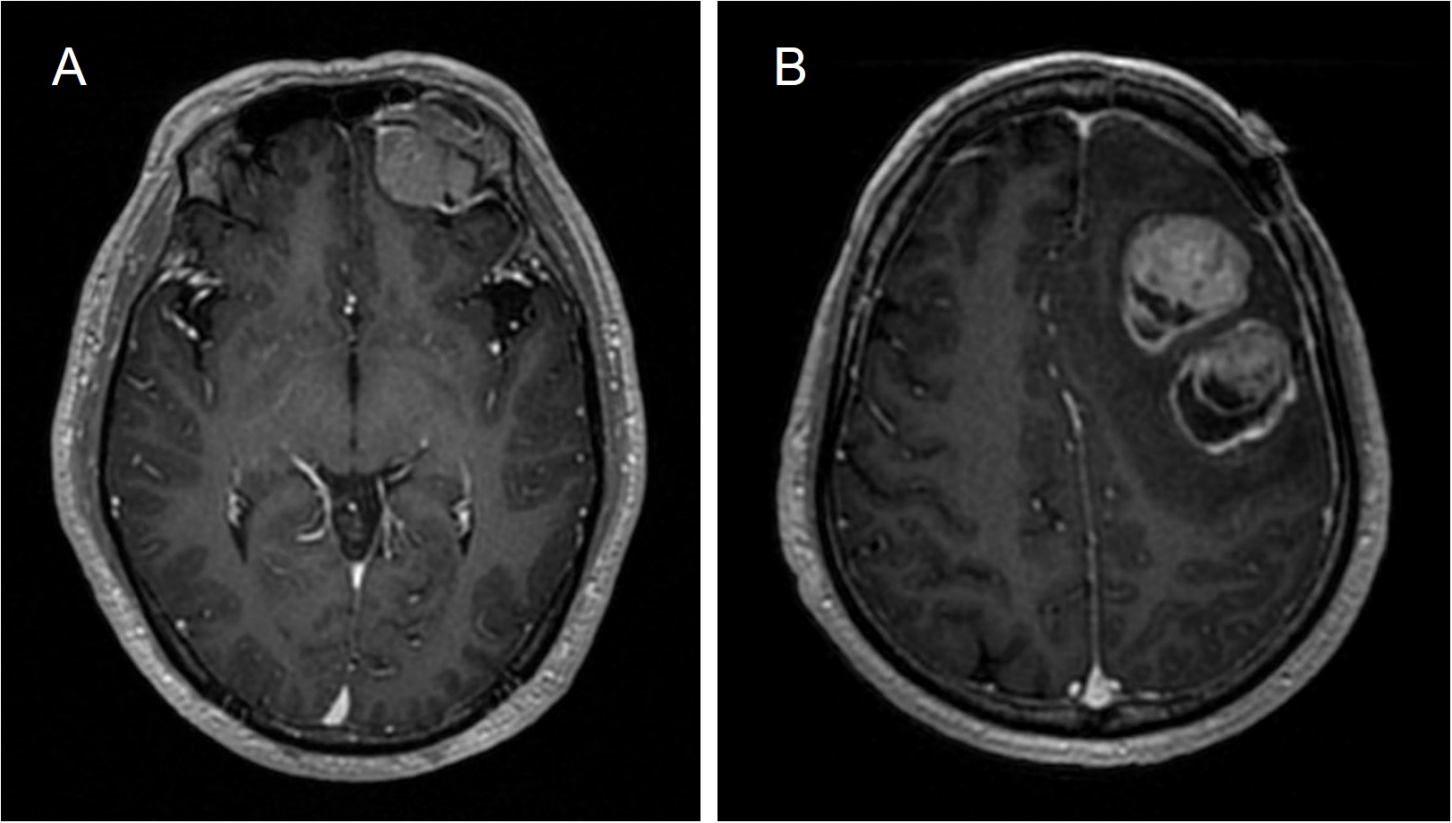
Brain MRI. **(A)** Initial primary tumor. A heterogeneously enhancing tumor on the left frontal lobe (32 × 29 × 31 mm^3^) with an associated mass effect. **(B)** Metastasis. Three well-defined, heterogeneously enhanced tumors in the left upper frontal lobe with found (39 ×31 ×42 mm^3^, 34 ×34 × 31 mm^3^, 13 ×8 × 14 mm^3^) with associated mass effect, with surrounding edema.

The initial magnetic resonance imaging (MRI) of the brain revealed a heterogeneously enhanced tumor on the left frontal lobe with an associated mass effect, and the enhanced MRI scan showed a dural tail sign (**Figure 1**). The boundary of the lesion was clear and no surrounding edema was seen. Magnetic resonance spectroscopy (MRS) of the complete lesion showed no obvious change in the NAA (N-acetyl aspartate) peak, and the Cho (choline) peak was not increased. The initial diagnosis was primary brain tumor but metastatic brain tumor could not be excluded. Preoperative systemic examinations, including computed tomography (CT), MRI, and positron emission tomography (PET)-CT also indicated no extracranial tumor. The patient underwent a left frontal craniotomy and complete tumor excision.

**Figure 2:**
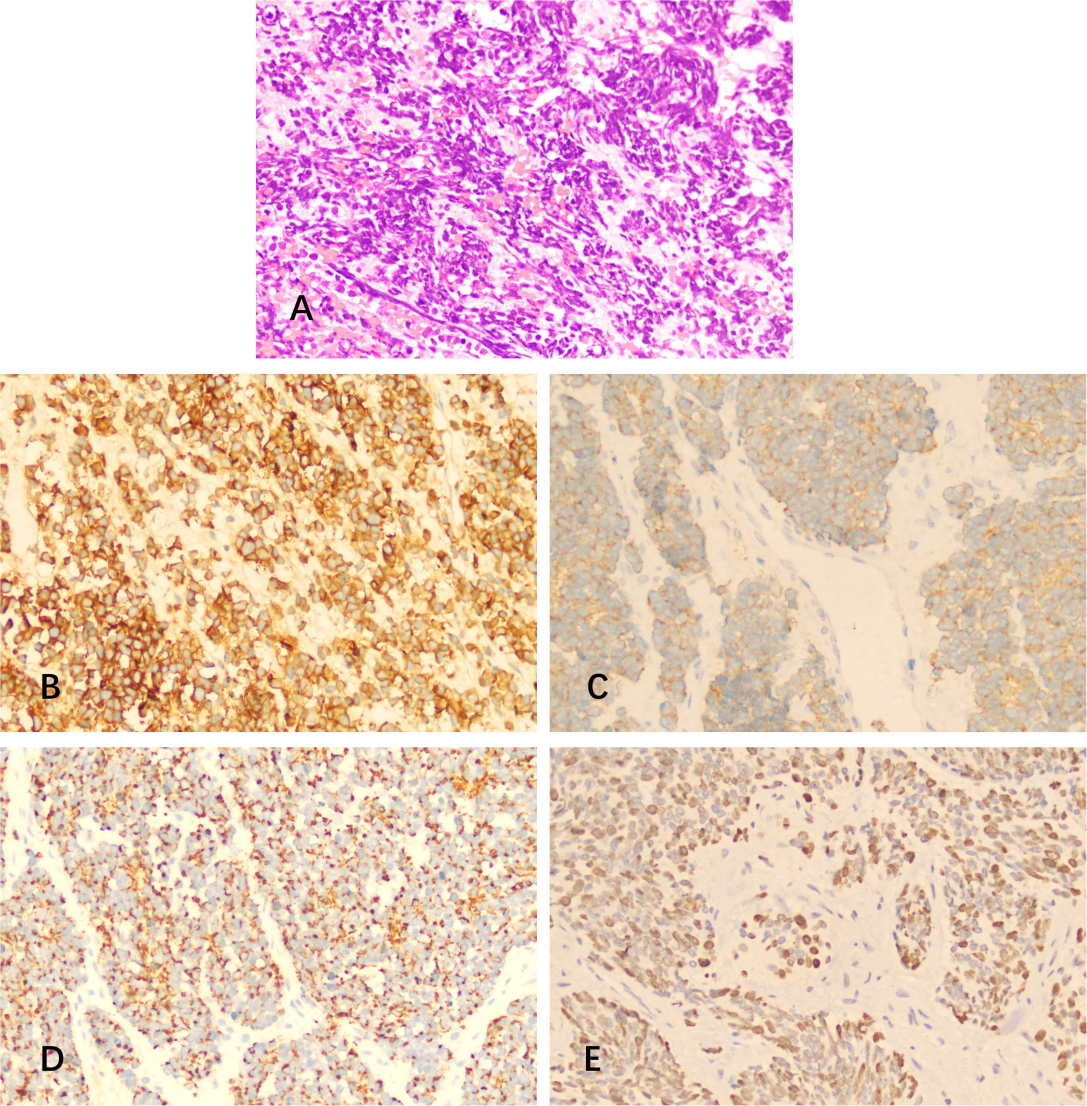
Hematoxylin and eosin (H&E) staining and immunostaining of specimens from the resected mass. **(A)** H&E revealed medullary small cells with scant cytoplasm, hyperchromatic nuclei, and numerous mitoses. **(B-E)** Immunohistochemistry showed positivity for **(B)** CD56, **(C)** Syn, **(D)** CK, and **(E)** Ki-67.

Histopathological analysis of the excised tissue showed medullary small cells with scant cytoplasm, hyperchromatic nuclei, and numerous mitoses (**Figure 2**). Dilated congested interstitial blood vessels with hemorrhage and meningeal invasion were also observed. Immunohistochemistry showed the presence of CD56, Syn, CK, and Ki-67 (30%), but TTF-1, GFAP, Bcl-6, MUM-1, C-Myc, Vimentin, P40, P53, CK7, CD3, CD5, CD20, CD79a, CD10, and CD23 were absent. Therefore, primary intracranial SCC was diagnosed. The patient subsequently underwent brain radiotherapy (30 grays in 30 fractions) and received 6 cycles of an EP (etoposide + cisplatin) chemotherapy regimen.

One year later the patient was readmitted to our department with neurological symptoms of slurred speech and slow movements. Brain MRI revealed 3 well-defined, heterogeneously enhanced tumors in the left upper frontal lobe with associated mass effect, surrounding edema, thickened meningeal enhancement, and the dural tail sign (**Figure 1**). MRS detected a significantly heightened Cho peak and a normal NAA peak. Ultimately, the tumor was excised through fluorescence-guided microsurgery.

**Figure 3:**
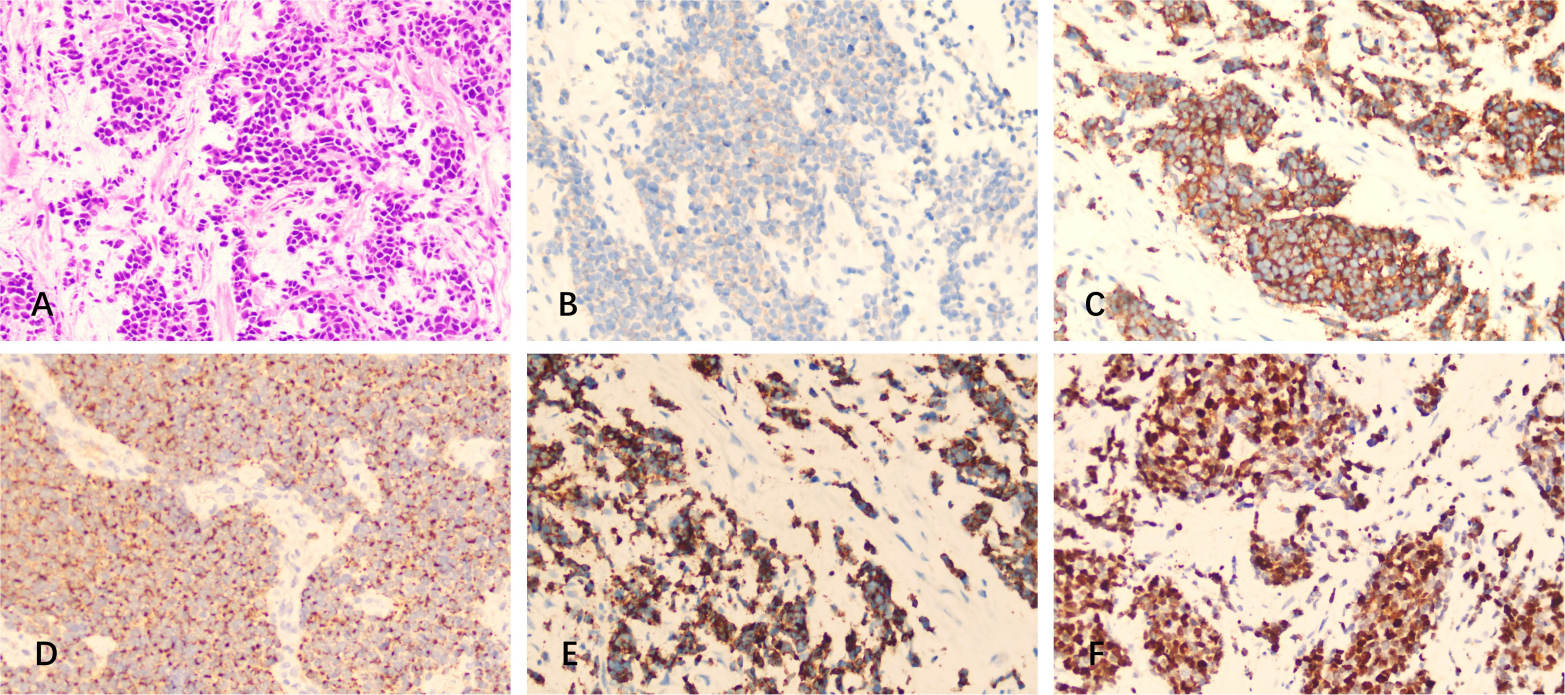
H&E staining and immunostaining of specimens from the resected mass (metastasis). **(A)** H&E staining. **(B-F)** Immunohistochemistry showed positivity for **(B)** CD56, **(C)** Syn, **(D)** CK, **(E)** CgA, and **(F)** Ki-67.

Immunohistochemical examination of the excision displayed the same pattern as the previous course, i.e., positive for CD56, Syn, CK, CgA, Ki-67 (90%), and negative for CK7, TTF-1, NSE, Napsin A, and S-100 (**Figure 3**). This indicated the recurrence of the intracranial SCC in the left frontal lobe.

Chromosomal copy number variations and amplifications were identified via next-generation sequencing. The following were detected: deletions of regions within chromosomes 10, 3-3, 7p22.3-p14.3, 9p24.3-q13, 10-10, 11-11, 12-12, 13q11-q14.2, 16-16, Xp22.33-Xq28, and Yq11.223-Yq12; and a major amplification in chromosomes 9q13, 1q12-q44, 5-5, 6-6, 7p14.3-p11.2, 18-18, 19-19, 20-20, and 22q11.1-q13.33.

The patient underwent radiotherapy (30 grays in 10 fractions) and a boost of 20 grays in 10 fractions to the tumor bed and received EP (etoposide 120 mg/m^2^ + cisplatin 100 mg/m^2^) chemotherapy after the second resection. Clinical examinations and imaging studies administered every 6 months after therapy excluded local and distant recurrence. The patient remained systemically well, and no extracranial disease was found by serial imaging (CT, MRI) at the 2-year follow-up.

## Discussion

EPSCC is an uncommon, highly aggressive malignancy. Primary intracranial SCC is extremely rare with only 8 reported cases (3–10). The prognosis of EPSCC is similar to that of SCLC, with a 5-year survival rate from 10% to 15% and a median overall survival of 9 to 15 months (11, 12). A small proportion of patients enjoy a prolonged disease-free interval or were even cured with aggressive combined-modality therapy (13).

A diagnosis of EPSCC is often challenging. Although immunohistochemistry may be helpful, EPSCC is mainly diagnosed by morphological characteristics that include round to spindle-shaped small cells with dense nuclei, granular nuclear chromatin, absent or inconspicuous nucleoli, and scant cytoplasm (14). The present case was also diagnosed based on the typical morphological features of SCC, and the neuroendocrine tumor marker Syn and CD56 were also found by immunohistochemistry.

The immunophenotype of EPSCC is highly heterogeneous. TTF-1 is present in up to 90% of pulmonary small cell carcinomas, and might be helpful in differentiating EPSCC from pulmonary tumors (15). The rates of TTF-1 positivity in EPSCC have been various (42% of 50 cases; 43.8% of 16 cases) (10, 16), with one report of 80% in 15 patients (17). Furthermore, TTF-1 positivity is a strong indicator of pulmonary-originated brain metastatic tumor, with an estimated incidence of 95% (18). Although in some cases TTF-1 negativity was not a determinate factor to distinguish pulmonary from extrapulmonary SCCs, in the present case TTF-1 negativity made a pulmonary origin less likely. The diagnosis of EPSCC was further confirmed by the lack of extracranial tumors in the follow-up CT, MRI, and PET/CT series.

A similar case was recently reported of a 68-year-old man with a primary intracranial SCC who developed no extracranial tumors in the year after tumor resection, based on CT and MRI scans (6). However, the immunohistochemical staining showed strong TTF-1 positivity, and it is possible that he had an occult lung primary tumor that was below the detection limits of the CT and MRI, or the tumor had originated from carcinogenesis in the tissues surrounding the former glioma site.

Cytogenetic analysis is a promising method to distinguish between pulmonary and extra-pulmonary disease, by detecting the loss of chromosome 10q and deletions in chromosome 13. These were recently emphasized as the origin of pulmonary disease (19). The loss of chromosome 3p,10q and deletion in chromosome 13 are more common in pulmonary SCC than in EPSCC (20). Yet, there is no united cytogenetic pattern that can definitively separate primary from metastatic, or pulmonary from EPSCC. In the present case, deletion of chromosomes 10, 3-3, q13, 10-10,13q11-q14.2 were confirmed. None of the identified amplification of chromosomes was associated with primary intracranial or extracranial SCC in our patient.

Due to the rarity of primary intracranial SCC, there has been limited investigation of optimal management strategies. The current clinical management of primary intracranial SCC is generally similar to that of pulmonary SCC or EPSCC, that is, combined surgery, chemotherapy and radiotherapy. The combination of cisplatin and etoposide (EP) is commonly used in chemotherapy for primary intracranial SCC. It is noteworthy that results of phase III clinical trials have suggested that immune checkpoint inhibitors have great potential when combined with chemotherapy in SCLC. In patients with extensive-stage SCLC, combining atezolizumab with chemotherapy was associated with significantly longer overall survival (21), and the CASPIAN trial also reported that adding durvalumab to the standard chemotherapy (EP) improved overall survival (22). Moreover, in the updated analysis of CASPIAN, 3 times more patients who underwent durvalumab plus EP were alive at 3 years compared with patients who received EP alone (23).

## Conclusion

Primary intracranial SCC is highly aggressive and extremely rare. Further studies are needed to explore optimal treatment strategies for SCC. Immune checkpoint inhibitors are promising strategies in this rare tumor type, and are currently under evaluation in phase III human clinical trials.

## Author contributions

YS and YZ contributed to writing this article and to the literature review, and RL and DC were responsible for immunohistochemical image data acquisition. ZY and WZ supervised the writing and revision of the manuscript. All authors contributed to the article and approved the submitted version.

## References

[1] Gazdar AF Bunn PA Minna JD. Small-cell lung cancer: what we know, what we need to know and the path forward. Nat Rev Cancer (2017) 17(12):725–37. doi: 10.1038/nrc.2017.87 29077690

[2] Lee SS Lee J-L Ryu H-M Chang HM Kim TW Kim WK. Extrapulmonary small cell carcinoma: single center experience with 61 patients. Acta Oncol (Stockholm Sweden) (2007) 46(6):846–51. doi: 10.1080/02841860601071893 17653910

[3] Galanis E Frytak S Lloyd RV. Extrapulmonary small cell carcinoma. Cancer (1997) 79(9):1729–36. doi: 10.1002/(SICI)1097-0142(19970501)79:9<1729::AID-CNCR14>3.0.CO;2-# 9128989

[4] Hueser CN Nguyen NC Osman M Havlioglu N Patel AJ. Extrapulmonary small cell carcinoma: involvement of the brain without evidence of extracranial Malignancy by serial PET/CT scans. World J Surg Oncol (2008) 6:102. doi: 10.1186/1477-7819-6-102 18817561 PMC2564932

[5] Noonan C James M. A case of isolated small cell carcinoma of the brain. Acta Oncol (Stockholm Sweden) (2017) 56(8):1133–5. doi: 10.1080/0284186X.2017.1290273 28562202

[6] Yang J Wang L Yao Y Liu J Yang J. Small cell carcinoma of the brain without apparent extracranial origin in the same intracranial region one year following resection of Malignant glioma. Int J Clin Exp Pathol (2021) 14(8):875–80. PMC8414428 34527130

[7] Terada T. Small cell carcinoma of the brain without extracranial involvement by serial CT, MRI and PET. Int J Clin Exp Pathol (2010) 3(3):323–7. PMC2836511 20224732

[8] Zhang SCai Q Fan L Zhang R Zhao Y Wu G. Primary intracranial small cell carcinoma: a case report and review of the literature. Onkologie (2013) 36(7-8):428–31. doi: 10.1159/000353566 23921762

[9] Chionh F Azad A Lee C. Small cell carcinoma of unknown primary presenting with disease confined to the central nervous system. Acta Oncol (Stockholm Sweden) (2009) 48(2):317–8. doi: 10.1080/02841860802311817 18752083

[10] Cheuk W Kwan MY Suster S Chan JK. Immunostaining for thyroid transcription factor 1 and cytokeratin 20 aids the distinction of small cell carcinoma from Merkel cell carcinoma, but not pulmonary from extrapulmonary small cell carcinomas. Arch Pathol Lab Med (2001) 125(2):228–31. doi: 10.5858/2001-125-0228-IFTTFA 11175640

[11] Haider K Shahid RK Finch D Sami A Ahmad I Yadav S. Extrapulmonary small cell cancer: a Canadian province’s experience. Cancer (2006) 107(9):2262–9. doi: 10.1002/cncr.22235 16998932

[12] Wong YN Jack RH Mak V Henrik M Davies EA. The epidemiology and survival of extrapulmonary small cell carcinoma in South East England, 1970-2004. BMC Cancer (2009) 9:209. doi: 10.1186/1471-2407-9-209 19563623 PMC2709640

[13] Berniker AV Abdulrahman AA Teytelboym OM Galindo LM Mackey JE. Extrapulmonary small cell carcinoma: imaging features with radiologic-pathologic correlation. Radiographics: Rev Publ Radiol Soc North America Inc (2015) 35(1):152–63. doi: 10.1148/rg.351140050 25590395

[14] Frazier SR Kaplan PA Loy TS. The pathology of extrapulmonary small cell carcinoma. Semin Oncol (2007) 34(1):30–8. doi: 10.1053/j.seminoncol.2006.11.017 17270663

[15] Verset L Arvanitakis M Loi P Closset J Delhaye M Remmelink M. TTF-1 positive small cell cancers: Don’t think they’re always primary pulmonary! World J Gastrointest Oncol (2011) 3(10):144–7. doi: 10.4251/wjgo.v3.i10.144 PMC3205113 22046491

[16] Agoff SN Lamps LW Philip AT Amin MB Schmidt RA True LD. Thyroid transcription factor-1 is expressed in extrapulmonary small cell carcinomas but not in other extrapulmonary neuroendocrine tumors. Modern Pathol (2000) 13(3):238–42. doi: 10.1038/modpathol.3880044 10757334

[17] Kaufmann O Dietel M. Expression of thyroid transcription factor-1 in pulmonary and extrapulmonary small cell carcinomas and other neuroendocrine carcinomas of various primary sites. Histopathology (2000) 36(5):415–20. doi: 10.1046/j.1365-2559.2000.00890.x 10792482

[18] Chuang W-Y Yeh C-J Chu P-H Liao C-C Wu C-T Chuang C-C. Expression of thyroid transcription factor-1 in brain metastases: a useful indicator of pulmonary origin. J Clin Neurosci (2008) 15(6):643–6. doi: 10.1016/j.jocn.2007.03.017 18413286

[19] Testa JR Liu Z Feder M Bell DW Balsara B Cheng JQ. Advances in the analysis of chromosome alterations in human lung carcinomas. Cancer Genet Cytogenet (1997) 95(1):20–32. doi: 10.1016/S0165-4608(96)00337-8 9140450

[20] Welborn J Jenks H Taplett J Walling P. High-grade neuroendocrine carcinomas display unique cytogenetic aberrations. Cancer Genet Cytogenet (2004) 155(1):33–41. doi: 10.1016/j.cancergencyto.2004.03.001 15527900

[21] Horn L Mansfield AS Szczęsna A Havel L Krzakowski M Hochmair MJ. First-line atezolizumab plus chemotherapy in extensive-stage small-cell lung cancer. New Engl J Med (2018) 379(23):2220–9. doi: 10.1056/NEJMoa1809064 30280641

[22] Paz-Ares L Dvorkin M Chen Y Reinmuth N Hotta K Trukhin D. Durvalumab plus platinum-etoposide versus platinum-etoposide in first-line treatment of extensive-stage small-cell lung cancer (CASPIAN): a randomised, controlled, open-label, phase 3 trial. Lancet (London England) (2019) 394(10212):1929–39. doi: 10.1016/S0140-6736(19)32222-6 31590988

[23] Paz-Ares L Chen Y Reinmuth N Hotta K Trukhin D Statsenko G. Durvalumab, with or without tremelimumab, plus platinum-etoposide in first-line treatment of extensive-stage small-cell lung cancer: 3-year overall survival update from CASPIAN. ESMO Open (2022) 7(2):100408. doi: 10.1016/j.esmoop.2022.100408 35279527 PMC9161394

